# Profiling the Influence of Gene Variants Related to Folate-Mediated One-Carbon Metabolism on the Outcome of In Vitro Fertilization (IVF) with Donor Oocytes in Recipients Receiving Folic Acid Fortification

**DOI:** 10.3390/ijms231911298

**Published:** 2022-09-25

**Authors:** Arturo Reyes Palomares, Maximiliano Ruiz-Galdon, Kui Liu, Armando Reyes-Engel, Kenny A. Rodriguez-Wallberg

**Affiliations:** 1 Laboratory of Translational Fertility Preservation, Department of Oncology and Pathology, Karolinska Institutet, 17164 Stockholm, Sweden; 2 IVF Unit, Instituto de Fertilidad Clínica Rincón, 29730 Malaga, Spain; 3 Department of Biochemistry and Molecular Biology, Faculty of Medicine, University of Malaga, 29071 Malaga, Spain; 4 Clinical Analysis Service, Hospital Regional Universitario de Malaga, 29010 Malaga, Spain; 5 Shenzhen Key Laboratory of Fertility Regulation, Center of Assisted Reproduction and Embryology, The University of Hong Kong-Shenzhen Hospital, Shenzhen 518000, China; 6 Department of Obstetrics and Gynecology, Li Ka Shing Faculty of Medicine, The University of Hong Kong, Hong Kong 999077, China; 7 Section of Reproductive Medicine, Division of Gynecology and Reproduction, Karolinska University Hospital, Novumhuset Plan 4, 14186 Stockholm, Sweden

**Keywords:** one-carbon metabolism, IVF, ART, embryo viability, folic acid, *MTHFR*, *ABCB1*, *BHMT*, *SHMT1*, gene polymorphism, oocyte donation

## Abstract

Nutritional status and gene polymorphisms of one-carbon metabolism confer a well-known interaction that in pregnant women may affect embryo viability and the health of the newborn. Folate metabolism directly impacts nucleotide synthesis and methylation, which is of increasing interest in the reproductive medicine field. Studies assessing the genetic influence of folate metabolism on IVF treatments have currently been performed in women using their own oocytes. Most of these patients seeking to have a child or undergoing IVF treatments are advised to preventively intake folate supplies that restore known metabolic imbalances, but the treatments could lead to the promotion of specific enzymes in specific women, depending on their genetic variance. In the present study, we assess the influence of candidate gene variants related to folate metabolism, such as Serine Hydroxymethyltransferase 1 *SHMT1* (rs1979276 and rs1979277)*,* Betaine-Homocysteine S-Methyltransferase *BHMT* (rs3733890)*,* Methionine synthase reductase *MTRR* (rs1801394)*,* Methylenetetrahydrofolate reductase *MTHFR* (rs1801131 and rs1801133)*,* methionine synthase *MTR* (rs12749581)*,* ATP Binding Cassette Subfamily B Member 1 *ABCB1* (rs1045642) and folate receptor alpha *FOLR1* (rs2071010) on the success of IVF treatment performed in women being recipients of donated oocytes. The implication of such gene variants seems to have no direct impact on pregnancy consecution after IVF; however, several gene variants could influence pregnancy loss events or pregnancy maintenance, as consequence of folic acid fortification.

## 1. Introduction

Evolution of assisted reproductive technologies (ART) have led to a significant increase in robustness of in vitro fertilization (IVF) treatment outcomes. The increase in reproductive success has been achieved by adaptation to more personalized screenings and ovarian stimulation protocols, but mostly as a consequence of the development of highly efficient embryo culture systems and cryopreservation methods. Despite great advances, still there is a lack of knowledge concerning one of the most singular events in mammalian reproduction: embryo implantation. Limitations for accessing the local environment for monitoring and sampling, together with ethical concerns, have limited the availability of good models to study and gain knowledge about the embryo–maternal dialogue.

High-throughput technologies have enabled the development of comprehensive screenings for increasing embryo performance, as is the case in preimplantation genetic testing for aneuploidies (PGT-A) and the assessment of endometrial receptivity through analysis of transcriptomes and microbiomes [1,2]. These screenings are very promising and likely to increase IVF treatments’ success; however, these are performed in the two interacting cellular systems separately, i.e., the embryo or the uterus, and thus lack the gain of knowledge about the biological reality throughout the interaction. In addition, the interaction at the embryo–maternal interface is conditioned by a surrounding milieu subjected to environmental agents, and the nutritional status of pregnant women should be also considered. The folate mediated one-carbon metabolism (FOCM) represents one of the most widely studied gene-environment interactions in utero with implications for embryo development and health in offspring [3]. Folate metabolism regulation could vary as a consequence of diet, pharmacological imbalances and genetic predisposition. In particular, the impact of low dietary folates, low vitamin B12 and the presence of gene variants modifying enzyme activity in pregnant women are associated with elevated homocysteine (Hcy) levels with different health consequences on fetuses, including neural tube defects (NTD) and other congenital disorders [4,5]. Between the negative consequences associated with Hcy accumulation underly the disruption of Hcy recycling into methionine throughout the re-methylation pathway, which impedes the synthesis of S-adenosylmethionine (SAM). It is worth mentioning that SAM is the universal methyl donor that promotes de novo DNA methylation during embryo reprogramming and its production is mediated by folate cycle metabolism [6]. The addition of folic acid fortification in the diet of pregnant women drastically reduced the incidence of NTD in newborns, leading to different public health agencies worldwide recommending it for use in pregnant women [7,8]. As is routine during IVF treatments, patients are recommended to have folic acid supplies even before the initiation of the treatment, so the prevention is presumably reached, especially considering that Hcy levels are normalized in approximately 10 days [9]. FOCM disruption can also be due to gene variants affecting key enzymes, such as those concerning the *MTHFR* gene with strong influence on reproduction and embryo viability [10,11]. Folic acid intake could even have a genetic impact in a general population leading to the enrichment of risk genotypes associated with lower activity of *MTHFR* in the population [12]. Despite the demonstrated benefits of folic acid fortification in pregnant women, its widely extended use in diets as a supplement is a matter of concern [13]. Deficiencies in folate and vitamins in a diet and genetic variants leading to FOCM defective enzymes could modify the epigenetic landscape. Interestingly, a trend towards a lower methylation profile has been hypothesized to occur as a consequence of the negative feedback loop exerted by high levels of folate on *MTHFR* activity [14]. Prolonged folic acid supplies up to the second and third trimester could be responsible for methylome changes on promotor and gene bodies related to neurodevelopment [15]. These patterns might be associated with better cognitive performance in children [15,16]. However, current studies have analyzed different tissues and thus it is still unknown to what extent there is a broad impact on the embryo and any potential long-term effects on newborns. 

Genetic background is probably one of the most determinant sources of variability and can be a powerful tool to gain knowledge about reproductive biological processes. It has been recently reported that Western lifestyles are compromising reproductive success in the European population through cumulative concentration of detrimental gene variants in reproductive terms [17]. The study of gene variants and their association with differences in reproductive outcomes can lead to a comprehensive understanding of infertility factors affecting different stages of conception [18,19,20,21]. Thus, we consider that studies attempting to clarify the genetic influence on reproduction outcome are needed in order to assess their efficacy, but also to increase follow-up and safety. The present study aims to assess the impact of gene variants related to FOCM on IVF outcomes of patients receiving folic acid supplementation. We investigated the impact of selected candidate gene variants on pregnancy consecution and maintenance in a pregnancy receptivity model of women receiving IVF using oocytes donated from fertile young donors.

## 2. Results

A total of 236 women underwent the current study, being genotyped for selected candidate gene polymorphisms involved in folate metabolism (Table 1). When allelic and genotype distributions of the study group were compared with a reference population of a similar genetic background (Iberian population), there were no differences in genotype and allelic distributions, only a higher representation of C allele observed in patients for *SHMT1* (*rs1979277*) [*p*-value = 0.036] (Table 2). 

Comparison between the two largest cohort groups, that is recipients that achieved a positive pregnancy test versus those who did not, showed a compliance of HW equilibrium in all the gene variants except for the group of recipients that become pregnant in the rs1045642 *ABCB1* (*p*-value = 0.018) and rs2071010 *FOLR1* (*p*-value = 0.034) gene polymorphisms (Table 2).

The general overview of treatments did not show differences in age and number of embryos transferred between recipients grouped by genotype (Table S1). Comparison of treatment outcomes did not reflect a relevant genetic influence except for the variant rs12749581 *MTR* in early biochemical pregnancy losses and the rs1045642 *ABCB1* with late miscarriages events that their incidence differed between recipients grouped by genotypes. 

Logistic regression analyses were computed to test prediction of clinical outcomes, such as no pregnancy, clinical pregnancy, ongoing pregnancy, biochemical pregnancy loss (BPL) and miscarriage events under different genetic models that could approximate a more realistic biological reality. After being adjusted for age and number of embryos transferred, comparisons were established for the different inheritance models stratified according to the binary outcomes (i.e., clinically pregnant vs. non-pregnant). The overall comparison did not show differences in genotype frequencies for all the studied single nucleotide polymorphisms (SNPs) between clinical pregnant and non-pregnant. By contrast, the analysis of early pregnancy losses showed an increased incidence of BPL in heterozygous C/T for *SHMT1* (rs1979277) compared with homozygous C/C and no cases in the minor group of T/T (4.7%) (Table 3). However, the T allele of rs1979277 at *SHMT1* could be an influence in more advanced ongoing pregnancies with an increased representation of heterozygous and homozygous T alleles in non-pregnant recipients (*p*-value = 0.027). At these advanced pregnancy stages, the rs3733890 *BHMT* showed a benefit of maintaining pregnancy associated either to the dominant model for A allele carriers [OR 0.58 (0.34–0.98) (*p*-value = 0.042)] or for the over-dominant model corresponding to heterozygous A/G [OR 0.55 (0.32–0.95) (*p*-value = 0.031)]. 

Comparison of pregnancy loss outcomes in different SNPs did only provide significant associations for the *ABCB1* (rs1045642), as observed in the general overview of the outcomes distributed by genotypes. The logistic regression model showed an increased risk of miscarriage for the T allele with a total absence of miscarriages in the recipients homozygous for CC (*p*-value = 0.010).

## 3. Discussion

The gene variants associated with the folate one-carbon metabolism did not influence pregnancy outcomes in women recipients of IVF/ICSI treatments undertaking folic acid fortification regime. Despite that, recipients were grouped by genotype, but this did not show any differences in pregnancy success, and *ABCB1* and *MTR* gene polymorphisms showed a potential association with early pregnancy losses and miscarriages. In addition, logistic regression analysis uncovered a further association with *SHMT1* and *BHMT* gene variants. The impact of these variants on IVF outcomes has not been previously reported and could provide new details about folate intake suitability for pregnancy establishment and maintenance.

The *ABCB1* gene is expressed in the placenta during the whole gestational period, being that protein levels increase 44.8 times higher during the early stage compared to the term pregnancy placenta [22,23]. The role of *ABCB1* is not fully understood but it has been hypothesized to act as a protective mechanism to xenobiotic and drugs present in the maternal blood, but also to endogenous and exogenous components especially relevant during this sensitive period [24]. Very interestingly, previous findings suggested an association between T alleles and poor outcomes which slightly resembled our results; i.e., there was observed an increased incidence of recurrent miscarriages in pregnant women [25]. In addition, an increased incidence of congenital disorders at birth has been reported in women receiving folic acid during pregnancy [26]. Lower expression and activity have been associated with the T allele [27], which could disrupt the efficiency of the hypothesized protective mechanism of *ABCB1* and, despite the benefits of folic acid, interact with other drugs used during IVF treatments. The variable expression of *ABCB1* in the placenta throughout pregnancy could be linked to the biphasic response to folic acid supplementation observed during early pregnancy establishment [28]. In other studies, the use of folic acid in T allele carriers reduced the risk for developing heart congenital disorders in offspring at risk [29]. This would indicate that perhaps the use of periconceptional folic acid should be personalized according to the specific risk to the fetus dependent on the carrier status of the mother. 

The functional impact of polymorphism rs12749581 in the *MTR* gene is still unknown to date and also makes difficult the very low frequency of the A allele only present in heterozygous recipients. Some other variants in *MTR* could modify S-adenosyl methionine (SAM) levels and influence methylation activity [30]. Variations in *MTR* in pregnant women have shown a clinical association to NTD, Down syndrome and other congenital disorders such as non-syndromic cleft palate [30,31,32]. Despite the allele frequency being very low, we decided to maintain the results observed in the group of heterozygous recipients (2%) that showed a highly increased incidence of biochemical pregnancy losses, and despite these surprising variations, these results should be taken with caution as it could be dependent on other unknown factors that may lead to bias.

Homozygous CC for *SHMT1* (rs1979277) presents lower folate and higher Hcy levels associated with the cytosolic isoform of *SHMT1* [33]. The negative effects on fertility associated with this metabolic profile could explain the C allele enrichment in our infertile cohort compared to the Iberian general population. However, according to our results under folic acid fortification, the T allele seems to have a negative influence on pregnancy establishment and maintenance. T alleles induce a lower expression of *SHMT1* in the uterus and the ovary (GTEx portal, Analysis Release V8 (dbGaP Accession phs000424.v8.p2)). Rebekah et al. found an increased risk of NTD disease associated with maternal T allele transmission [34], and a recent study also showed an association between the T allele at *SHMT1* rs1979277 and fetal growth restriction [35]. Other authors suggested that the lower activity of *SHMT1* associated with the T allele may lead to folate retention in the cytoplasm that could ensure its availability, thus reducing the risks of cleft palate associated with a deficiency of folate [36]. These previous studies did not specify the folic intake of the study groups involved; our cohort preventively used folic acid, which may have had a negative impact on the pregnancies of T allele recipients. It is worth mentioning that our study did not consider the potential paternal or maternal transmission towards the embryo, which could also imply differences. 

Betaine functions as a methyl donor and the substrates of the *BHMT* enzyme on the Hcy remethylation pathway are converted to Dimethylglycine (DMG) [37]. DMG is good marker of betaine utilization and inhibits *BHMT* activity [37,38]. The rs3733890 gene polymorphism induces a missense change in the aminoacidic sequence of the enzyme, which theoretically leads to an increased affinity for Hcy and no variation in the Michaelis constant for betaine [39,40,41]. No variations on total Hcy levels induced by this polymorphism have been reported [42,43,44]; however, during early pregnancy, transformation of betaine to DMG is precisely lower in carrying the A allele and with a high folate status [45]. Colomina et al. speculated that under low folate MTR activity would be reduced and lead to BHMT upregulation, also with the existence of a potential inhibition of *BHMT* by high folate levels [45]. In our cohort there is no presumptive representation of low folate pregnant women due every patient was recommended folic acid intake and, according to our results, less favorable conditions were seen in homozygous recipients for the common variant GG. Despite the lack of statistical significance, during the early pregnancy stages, recipients carrying the A allele showed higher implantation rates than homozygous GG (43.13% vs. 34.02%). It could be hypothesized that *BHMT* enzymatic inhibition carried out by high folic acid and DMG levels was stronger in homozygous GG during the exceptional metabolic condition observed in early pregnancies. The impact of this polymorphism has been also studied in more advanced stages of pregnancy, being the A allele associated with placental abruption in patients receiving folic acid fortification presented as relatively high, but showed lower blood levels of folate compared to control [46]; it worth mentioning that placental abruption was considered after the 20th week of gestation for the period when folic acid fortification might be already interrupted. The maternal genotype condition of this polymorphism and folic status have been also associated with NTD [47]. Liu et al. observed an increased risk of NTD associated with the A allele in women that did not take folic acid during pregnancy [48]. The variable metabolic conditions throughout pregnancy, folate status and genotype at rs3733890 gene polymorphism should be investigated for adapting more personalized support at either early pregnancy establishment or embryo development. 

The influence of *MTHFR* gene polymorphisms on pregnancy consecution and embryo viability has been previously reported [10,11,49]. Notwithstanding, in the same line than previous studies, our results did not show significant differences in IVF outcomes promoted by differences in maternal genotypes at C677T and A1298C *MTHFR* gene polymorphisms [50]. An initial assessment during a fertility consultation comprises a general overview of the nutritional status of IVF patients and most of them are recommended to initiate folate fortification, which seem to spare folate levels in IVF patients [51]. The maintenance of a threshold in folate levels impedes genetically originated deficiencies in *MTHFR* to influence IVF reproductive success without increasing the risk of genetic abnormalities [51,52]. However, our results suggest that other maternal genetic variants affecting participating enzymes within the FOCM are different than *MTHFR*, which could influence reproductive options in recipients of donated oocytes.

The impact of folic acid intake could vary during embryo development and pregnancy progression, making it necessary to assess adequate folate dosages and timing. In addition, different doses and folate forms, such as methylfolate, are effectively used as alternatives to reduce Hcy levels, especially when these are still persistently high after use of the standard 400 μg/day folic acid. How these different strategies to control FOCM metabolism could be affected throughout pregnancy is still unknown. As some authors propose, new studies should include more comprehensive (epi)genomic assessments [53]. Our study suggests that the use of folic acid fortification in IVF patients may stress the FOCM to different enzymes within the pathway and this could have potential implications for pregnancy maintenance. The strength of our study relies on the control of fertility parameters with respect to oocyte biology that could impair implantation and pregnancy through the use of good quality oocytes from donors. Limitations of our study include the lack of measures of the biochemical parameters of FOCM. Additionally, although the standard dose of 400 μg/day folic acid was used in the study, we cannot ignore that other drugs used during IVF treatment could interfere as well on FOCM. Our study provides new information about potential metabolic changes during the embryo–maternal dialogue, a niche topic with very limited accessibility for experimental studies. New comprehensive pharmacogenetic and epigenetic approaches over different stages of development and pregnancy as well as those addressed to assess the health status of children born under different conditions would be useful to ascertain the benefits and risks of folic acid supplements.

## 4. Material and Methods

### 4.1. Study Design and Patient Inclusion

Caucasian European women aged 18–45 years undergoing their first IVF treatment as recipients of embryos generated using donated oocytes were recruited for the present study. All women included received at least two good quality embryos on day 2 or day 3 in fresh cycles and were prescribed 400 μg/day folic acid from the start of the treatment. Patients presenting any known conditions that could compromise implantation, such as uterine factor infertility, abnormal endometrial thickness or suspected hydrosalpinx were excluded, as well as women with body mass index (BMI) > 30 Kg/m^2^ and women with a partner presenting with severe male factor. DNA isolation was performed through buccal swabs. All subjects provided signed informed consent to participate in the study. The study was approved by the ethics committee of the University of Malaga and Hospital Universitario Virgen de la Victoria, Malaga, Spain. 

### 4.2. Oocyte Retrieval, Insemination, Embryo Culture and Transfer

The donors included in the present study were all Caucasian European women. Following Spanish national guidelines, all donors were screened regarding physical and mental health, were aged 18–35 years, with an absence of known hereditary disorders, chromosome abnormalities or infectious diseases. 

After controlled ovarian stimulation, oocyte maturation was triggered by administering a GnRH analogue when at least three dominant follicles were more than 17 mm in size. Ovarian punction was programmed 35 h after GnRH analogue administration for subsequent oocyte retrieval. After mechanical denudation of cumulus cells, the mature oocytes metaphase II were microinjected through intracytoplasmic sperm injection (ICSI) using sperm of the male partner or donor. Fertilization and embryo morphological assessment were carried out for every day of incubation at 37 °C, 95% humidity, low oxygen atmosphere 5% (O_2_) and 6% (CO_2_). According to the Spanish Society for Studies on Reproductive Biology (ASEBIR), embryos were scored and then classified for transfer, and only cases compromising at least two good quality embryos defined by A or B were included [54]. Finally, embryo transfer was programmed on DAY + 2 or DAY + 3. 

### 4.3. Clinical Outcomes

Pregnancy was confirmed by serum or urine test after two consecutive measurements on DAY + 14 from the day of oocyte injection, as previously reported [18,21]. Clinical pregnancy (CP) was demonstrated by ultrasonographical evidence of a gestational sac around weeks 5 and 6. The maintenance of a viable pregnancy evidenced by heart beat activity after week 12 of gestation was indicated as an ongoing pregnancy (OP). Calculation of implantation rate (IR) was then established by the ratio between number of gestational sacs found and the number of embryos transferred. By contrast, the absence of a gestational sac and a decrease of serum β-hCG levels or lack of correspondence with gestational age was considered a biochemical pregnancy loss (BPL). Miscarriage (M) was considered in cases when a pregnancy occurred at a later stage, after the identification of a gestational sac and at most on the 20th week [55]. Ultrasound observation of a proper sac or signs of it outside the uterine cavity was considered an ectopic pregnancy [56]. Those women without a test determining pregnancy after the treatment were defined as nonpregnant. 

### 4.4. DNA preparation and Genotyping 

Genotyping was carried out as previously reported [21]. Genomic DNA was isolated from buccal swabs collected from the recipients and using the QIAmp DNA Mini Kit (Qiagen, Valencia, CA, USA). The genotyping process was outsourced to the Genetic and Proteomic services of Science and Technology School at the University of Pais Vasco (Bizkaia, Spain). A Taqman^®^ Open Array Genotyping System (Applied Biosystems, Foster City, CA, USA) was used to identify *SHMT1* (rs1979276 and rs1979277)*, BHMT* (rs3733890)*, MTRR* (rs1801394)*, MTHFR* (rs1801131 and rs1801133)*, MTR* (rs12749581)*, ABCB1* (rs1045642) and *FOLR1* (rs2071010). Through the use of Taqman Genotyper^®^ software (Applied Biosystems, Foster City, CA, USA), alleles were assigned. For every reaction, positive and negative controls were included for ensuring the quality and concordance of the results as well as for validation. A minimum of 80% calling rate was considered to assign genotypes in the SNP. 

### 4.5. Statistical Analysis

A nested case control study was performed to investigate the relationship between known gene polymorphisms influencing one carbon metabolism and IVF treatment outcomes. Progression of treatments depending on pregnancy success and evolution defined different group of patients. Outcomes were compared individually per polymorphism and according to allelic and genotypic composition. Assessment of potential deviation of genotype frequencies of the whole group were performed by comparing with an equivalent Caucasian population from 1000 Genome (Phase 3) reference [57]. 

Chi-squared tests were used to assess compliance with the Hardy–Weinberg (HW) equilibrium and pooled by genotype, and differences between age and number of embryos transferred were calculated by analysis of the variance (ANOVA). Potential association of gene polymorphisms with different outcomes such as IR, CP, BPL, M and OP was assessed by the Chi-squared test and Fisher’s exact tests. If there were any difference in reproductive outcomes between genotypes, a further logistic regression model was applied to compute odds ration with a 95% confidence interval. Age and number of embryos transferred were considered as potential confounders and treated for adjustment in logistic regression calculations.

In the absence of biological knowledge about the different genotype functional effects and possible inheritance, models were considered as recessive, dominant, codominant and over-dominant. Through SNPassoc under the R platform, Akaike’s information criteria (AIC) were used to select the fittest association for the different logistic regression models calculated. The *p*-value < 0.05 for the likelihood ratio test of association was considered as statistically significant either for the Chi-squared or Fisher’s exact test [58,59].

## Figures and Tables

**Table 1:** Detail and description of candidate gene variants related to folate mediated one-carbon metabolism pathway and selected for the present study.

Gen	Variation Sense	Variant Consequence	Allele Frequencies	Call Rate N (%)
*SHMT1 (rs1979276)*	C > T	3 prime UTR variant	C: 67% T: 33%	217/236 (91.95%)
*SHMT1 (rs1979277)*	C > T	Missense	C: 70% T: 30%	205/236 (86.86%)
*BHMT (rs3733890)*	G > A	Missense	G: 66% A: 34%	223/236 (94.49%)
*MTRR (rs1801394)*	A > G	Missense	A: 51% G: 49%	209/236 (88.56%)
*MTHFR (rs1801131)*	A > C	Missense	A: 73% C: 27%	220/236 (93.22%)
*MTHFR (rs1801133)*	C > T	Missense	C: 56% T: 44%	224/236 (94.92%)
*MTR (rs12749581)*	G > A	Missense	G: 99% A: 1%	219/236 (92.8%)
*ABCB1 (rs1045642)*	T > C	Missense	A: 46% T: 54%	222/236 (94.07%)
*FOLR1 (rs2071010)*	G > A	5 prime UTR variant	G: 94% A: 6%	222/236 (94.07%)

Note: Percentage Allele frequencies for European Iberian population from 1000 Genome (GRCh38.p13).

**Table 2:** Allele and genotype frequencies comparison between total study group and reference population * and the two largest groups of the cohort β-hCG (+) and β-hCG (−) recipients. Hardy–Weinberg equilibrium assessment.

Gene (Variant ID)	Alleles Genotypes	Patients n (Freq.)	Reference Population * n (Freq.)	^c^ *p* Value	β-hCG (+) n (Freq.)	β-hCG (−) n (freq.)	^d^ *p* Value
*SHMT1 (rs1979276)*	C	316 (0.73)	144 (0.67)	0.145	210 (0.73)	106 (0.73)	1
	T	118 (0.27)	70 (0.33)		78 (0.27)	40 (0.27)	
	C/C	115 (0.53)	48 (0.45)	0.343	77 (0.53)	38 (0.52)	0.942
	C/T	86 (0.4)	48 (0.45)		56 (0.39)	30 (0.41)	
	T/T	16 (0.07)	11 (0.10)		11 (0.08)	5 (0.07)	
*SHMT1 (rs1979277)*	C	317 (0.77)	149 (0.70)	^a^ 0.036	207 (0.78)	110 (0.76)	0.740
	T	93 (0.23)	65 (0.30)		59 (0.22)	34 (0.24)	
	C/C	121 (0.59)	51 (0.48)	0.101	80 (0.6)	41 (0.57)	0.883
	C/T	75 (0.37)	47 (0.44)		47 (0.35)	28 (0.39)	
	T/T	9 (0.04)	9 (0.08)		6 (0.05)	3 (0.04)	
*BHMT (rs3733890)*	G	304 (0.68)	141 (0.66)	0.600	187 (0.65)	117 (0.73)	0.093
	A	142 (0.32)	73 (0.34)		99 (0.35)	43 (0.27)	
	G/G	107 (0.48)	45 (0.42)	0.453	63 (0.44)	44 (0.55)	0.258
	A/G	90 (0.40)	51 (0.48)		61 (0.43)	29 (0.36)	
	A/A	26 (0.12)	11 (0.10)		19 (0.13)	7 (0.09)	
*MTRR (rs1801394)*	A	215 (0.51)	108 (0.51)	0.823	142 (0.52)	73 (0.5)	0.663
	G	203 (0.49)	106 (0.49)		130 (0.48)	73 (0.5)	
	A/A	56 (0.27)	28 (0.26)	0.965	36 (0.26)	20 (0.27)	0.619
	A/G	103 (0.49)	52 (0.49)		70 (0.51)	33 (0.45)	
	G/G	50 (0.24)	27 (0.25)		30 (0.22)	20 (0.27)	
*MTHFR (rs1801131)*	A	291 (0.66)	156 (0.73)	0.081	186 (0.65)	105 (0.68)	0.507
	C	149 (0.34)	58 (0.27)		100 (0.35)	49 (0.32)	
	A/A	103 (0.47)	55 (0.51)	0.060	65 (0.45)	38 (0.49)	0.823
	A/C	85 (0.39)	46 (0.43)		56 (0.39)	29 (0.38)	
	C/C	32 (0.15)	6 (0.06)		22 (0.15)	10 (0.13)	
*MTHFR (rs1801133)*	C	258 (0.58)	119 (0.56)	0.631	166 (0.58)	92 (0.57)	0.791
	T	190 (0.42)	95 (0.44)		120 (0.42)	70 (0.43)	
	C/C	77 (0.34)	30 (0.28)	0.326	50 (0.35)	27 (0.33)	0.966
	C/T	104 (0.46)	59 (0.55)		66 (0.46)	38 (0.47)	
	T/T	43 (0.19)	18 (0.17)		27 (0.19)	16 (0.2)	
*MTR (rs12749581)*	G	434 (0.99)	213 (0.99)	0.672	279 (0.99)	155 (0.99)	^b^ 1
	A	4 (0.01)	1 (0.01)		3 (0.01)	1 (0.01)	
	G/G	215 (0.98)	88 (0.97)	^b^ 0.678	138 (0.98)	77 (0.99)	^b^ 1
	G/A	4 (0.02)	3 (0.03)		3 (0.02)	1 (0.31)	
*ABCB1 (rs1045642)*	T	223 (0.5)	99 (0.46)	0.340	^e^ 140 (0.5)	83 (0.51)	0.752
	C	221 (0.5)	115 (0.54)		^e^ 142 (0.5)	79 (0.49)	
	T/T	64 (0.29)	25 (0.23)	0.574	^e^ 42 (0.3)	20 (0.25)	0.381
	T/C	95 (0.43)	50 (0.47)		^e^ 56 (0.4)	39 (0.48)	
	C/C	63 (0.28)	32 (0.30)		^e^ 43 (0.3)	20 (0.27)	
*FOLR1 (rs2071010)*	G	418 (0.94)	202 (0.94)	0.888	^e^ 268 (0.92)	150 (0.97)	^a^ 0.033
	A	26 (0.06)	12 (0.06)		^e^ 22 (0.08)	4 (0.03)	
	G/G	199 (0.90)	95 (0.89)	0.404	^e^ 126 (0.87)	73 (0.95)	^b^ 0.189
	G/A	20 (0.09)	12 (0.11)		^e^ 16 (0.11)	4 (0.05)	
	A/A	3 (0.01)	0 (0.00)		^e^ 3 (0.02)	0 (0.00)	

Note. Genotype and allele frequencies comparison with European population (in the left) and also between recipients with positive (β-hCG (+)) and negative (β-hCG (−)) pregnancy tests. Hardy–Weinberg equilibrium comparison (In the right). Results represent the *p*-value (Pearson) of Chi-squared Test and Freq. (Frequency). * (Frequency population of the 1000Genome Phase 3 (GRCh38.p13) for the selected gene variants). ^a^ *p* < 0.05; ^b^ Fisher’s exact test; ^c^ Patients vs. Reference Population; ^d^ β-hCG (+) vs. β-hCG (−); ^e^ Not compliant with HW [*ABCB1 rs1045642* (*p*-value = 0.018) and *FOLR1 rs2071010* (*p*-value = 0.034)].

**Table 3:** Logistic regression analysis of gene variants and the probability of achieving a defined clinical outcome.

Variant	Inheritance Model	Genotype	Clinical Outcome		OR (95% CI)	*p*-Value	AIC
			Ongoing Pregnant Freq. (%)	Non-pregnant Freq. (%)			
*BHMT* (*rs3733890*)	Codominant	G/G	47 (41.2%)	60 (55%)	1.00	0.082	313.5
		A/G	54 (47.4%)	36 (33%)	**0.53 (0.30–0.93)**		
		A/A	13 (11.4%)	13 (11.9%)	0.79 (0.33–1.88)		
	Dominant	G/G	47 (41.2%)	60 (55%)	1.00	0.042 ^a^	312.3
		A/G-A/A	67 (58.8%)	49 (45%)	**0.58 (0.34–0.98)**		
	Recessive	G/G-A/G	101 (88.6%)	96 (88.1%)	1.00	0.89	316.4
		A/A	13 (11.4%)	13 (11.9%)	1.06 (0.47–2.42)		
	Over-dominant	G/G-A/A	60 (52.6%)	73 (67%)	1.00	0.03 ^a^	311.8 ^b^
		A/G	54 (47.4%)	36 (33%)	**0.55 (0.32–0.95)**		
*SHMT1 (rs1979277)*	Codominant	C/C	70 (66%)	51 (51.5%)	1.00	0.086	288.2
		C/T	32 (30.2%)	43 (43.4%)	**1.91 (1.06–3.45)**		
		T/T	4 (3.8%)	5 (5%)	1.74 (0.44–6.84)		
	Dominant	C/C	70 (66%)	51 (51.5%)	1.00	0.027 ^a^	286.2 ^b^
		C/T-T/T	36 (34%)	48 (48.5%)	**1.89 (1.07–3.35)**		
	Recessive	C/C-C/T	102 (96.2%)	94 (95%)	1.00	0.65	290.9
		T/T	4 (3.8%)	5 (5%)	1.36 (0.35–5.22)		
	Over-dominant	C/C-T/T	74 (69.8%)	56 (56.6%)	1.00	0.039 ^a^	286.9
		C/T	32 (30.2%)	43 (43.4%)	**1.84 (1.03–3.29)**		
			Clinical pregnant Freq. (%)	BPL Freq. (%)			
*SHMT1 (rs1979277)*	Codominant	C/C	79 (62.2%)	1 (16.7%)	1.00	0.05	52.4
		C/T	42 (33.1%)	5 (83.3%)	**9.02 (1.01–80.56)**		
		T/T	6 (4.7%)	0 (0%)	0.00 (0.00–NA)		
	Dominant	C/C	79 (62.2%)	1 (16.7%)	1.00	0.029 ^a^	51.7
		C/T-T/T	48 (37.8%)	5 (83.3%)	7.91 (0.89–70.39)		
	Recessive	C/C-C/T	121 (95.3%)	6 (100%)	1.00	0.45	55.8
		T/T	6 (4.7%)	0 (0%)	0.00 (0.00–NA)		
	Over-dominant	C/C-T/T	85 (66.9%)	1 (16.7%)	1.00	0.016 ^a^	50.6 ^b^
		C/T	42 (33.1%)	5 (83.3%)	**9.73 (1.09–86.69**)		
			Ongoing Pregnant Freq. (%)	Miscarriage Freq. (%)			
*ABCB1* *(rs1045642)*	Codominant	T/T	31 (27.4%)	8 (53.3%)	1.00	0.0023 ^a^	89.8
		T/C	45 (39.8%)	7 (46.7%)	0.58 (0.19–1.79)		
		C/C	37 (32.7%)	0 (0%)	0.00 (0.00–NA)		
	Dominant	T/T	31 (27.4%)	8 (53.3%)	1.00	0.042 ^a^	95.8
		T/C-C/C	82 (72.6%)	7 (46.7%)	**0.32 (0.11–0.96)**		
	Recessive	T/T-T/C	76 (67.3%)	15 (100%)	1.00	8 × 10^4 a^	88.7 ^b^
		C/C	37 (32.7%)	0 (0%)	0.00 (0.00–NA)		
	Over-dominant	T/T-C/C	68 (60.2%)	8 (53.3%)	1.00	0.62	99.7
		T/C	45 (39.8%)	7 (46.7%)	1.32 (0.44–3.90)		

Note: Ongoing pregnancy was defined as confirmation of heartbeat after week 12 of gestation, clinical pregnancy was defined by ultrasound detection of a gestational sac at week 5–6 of gestation, biochemical pregnancy loss (BPL) was defined as a positive pregnancy serum test but no clinical pregnancy evidenced by ultrasound, and miscarriage was defined as the pregnancy loss occurring after ultrasonographic evidence of a clinical pregnancy. Different inheritance models were used to compare the possible associations between genotypes and clinical outcomes. AIC values were used to rank the fittest models which were selected by the lowest value of both. OR = odds ratio (significant in bold) with their respective CI = Confidence interval. ^a^ *p*-value < 0.05 (Chi-squared test) (Adjusted by age) and ^b^ lowest AIC.

## Data Availability

Not applicable.

## Supplementary Materials

The supporting information can be downloaded at: https://www.mdpi.com/article/10.3390/ijms231911298/s1.
