# Supplementary material for: Profiling the Influence of Gene Variants Related to Folate-Mediated One-Carbon Metabolism on the Outcome of In Vitro Fertilization (IVF) with Donor Oocytes in Recipients Receiving Folic Acid Fortification
Source: Int J Mol Sci. 2022 Sep 25;23(19):11298. doi: 10.3390/ijms231911298 (PMC9569987; doi:10.3390/ijms231911298)
Supplement: Supplementary file 1 [file ijms-23-11298-s001.zip › ijms-1937054-supplementary.pdf]

Table S1. IVF outcomes between recipients grouped by genotype.

Gene (Variant ID)	Genotype	Age (Mean±SD)	Nº ET (Mean±SD)	Implantation (%)	Clinical Pregnancy (%)	Biochemical Pregnancy Loss (%)	Miscarriage (%)	Ongoing Pregnancy (%)
SHMT1 (rs1979276)	C/C	40.83 ± 3.92	2.20 ± 0.48	99/253 (39.13)	75/115 (65.22)	2/77 (2.60)	9/75 (12.00)	65/115 (56.52)
	C/T	40.20 ± 5.06	2.20 ± 0.41	75/189 (39.68)	51/86 (59.30)	5/56 (8.93)	7/51 (13.73)	40/86 (46.51)
	T/T	40.38 ± 3.32	2.44 ± 0.63	20/39 (51.28)	11/16 (68.75)	0/11 (0.00)	1/11 (9.09)	9/16 (56.25)
		0.584	0.151	0.345	0.616	0.193[a]	0.918[a]	0.355
SHMT1 (rs1979277)	C/C	40.88 ± 3.94	2.18 ± 0.47	104/264 (39.39)	79/121 (65.29)	1/80 (1.25)	7/79 (8.86)	70/121 (57.85)
	C/T	39.91 ± 5.14	2.24 ± 0.43	63/168 (37.50)	42/75 (56.00)	5/47 (10.64)	7/42 (16.67)	32/75 (42.67)
	T/T	40.44 ± 3.36	2.22 ± 0.67	10/20 (50.00)	6/9 (66.67)	0/6 (0.00)	1/6 (16.67)	4/9 (44.44)
		0.325	0.691	0.552	0.393[a]	0.057[a]	0.272[a]	0.103[a]
BHMT (rs3733890)	G/G	40.72 ± 3.93	2.25 ± 0.52	82/241 (34.02)	59/107 (55.14)	4/63 (6.35)	9/59 (15.25)	47/107 (43.93)
	A/G	40.42 ± 4.47	2.23 ± 0.45	84/201 (41.79)	59/90 (65.56)	2/61 (3.28)	4/59 (6.78)	54/90 (60.00)
	A/A	41.23 ± 3.15	2.07 ± 0.27	26/54 (48.15)	19/26 (73.08)	0/19 (0.00)	4/19 (21.05)	13/26 (50.00)
		0.658	0.225	0.080	0.141	0.612[a]	0.108[a]	0.079
MTRR (rs1801394)	G/G	40.48 ± 3.60	2.26 ± 0.49	42/113 (37.17)	29/50 (58.00)	1/30 (3.33)	3/29 (10.34)	26/50 (52.00)
	A/G	40.83 ± 4.27	2.16 ± 0.41	84/222 (37.84)	66/103 (64.08)	4/70 (5.71)	7/66 (10.61)	55/103 (53.40)
	A/A	40.02 ± 4.59	2.25 ± 0.48	56/126 (44.44)	35/56 (62.50)	1/36 (2.78)	3/35 (8.57)	30/56 (53.57)
		0.508	0.280	0.407	0.767	0.868[a]	0.999[a]	0.985
MTHFR (rs1801131)	A/A	40.79 ± 3.81	2.19 ± 0.49	88/226 (38.94)	62/103 (60.19)	3/65 (4.62)	3/62 (4.84)	56/103 (54.37)
	A/C	40.19 ± 5.01	2.22 ± 0.45	71/189 (37.57)	53/85 (62.35)	3/56 (5.36)	7/53 (13.21)	46/85 (54.12)
	C/C	40.59 ± 4.61	2.19 ± 0.40	33/70 (47.14)	21/32 (65.63)	1/22 (4.55)	4/21 (19.05)	14/32 (43.75)
		0.658	0.887	0.362	0.852	1[a]	0.081[a]	0.546
MTHFR (rs1801133)	C/C	40.60 ± 5.02	2.20 ± 0.43	77/169 (45.56)	49/77 (63.64)	1/50 (2.00)	5/49 (10.20)	40/77 (51.95)
	C/T	40.25 ± 3.88	2.22 ± 0.44	80/231 (34.63)	62/104 (59.62)	4/66 (6.06)	6/62 (9.68)	55/104 (52.88)
	T/T	41.39 ± 3.44	2.16 ± 0.53	38/95 (40.00)	26/44 (59.09)	2/28 (7.14)	2/26 (7.69)	23/44 (52.27)
		0.328	0.741	0.086	0.831	0.477[a]	0.999[a]	0.990
MTR (rs12749581)	G/G	40.52 ± 4.14	2.21 ± 0.46	188/474 (39.66)	133/215 (61.86)	5/138 (3.62)	16/133 (12.03)	111/215 (51.63)
	A/G	38.00 ± 10.30	2.50 ± 0.58	1/10 (10.00)	1/4 (25.00)	2/3 (66.67)	0/1 (0.00)	1/4 (25.00)
	A/A	N/A	N/A	N/A	N/A	N/A	N/A	N/A
		0.247	0.205	0.097[a]	0.301[a]	0.006[a.b]	1.00	0.360[a]
ABCB1 (rs1045642)	C/C	41.10 ± 3.54	2.19 ± 0.47	59/138 (42.75)	39/63 (61.90)	4/43 (9.30)	0/39 (0.00)	37/63 (58.73)
	C/T	40.78 ± 3.94	2.23 ± 0.45	70/212 (33.02)	54/95 (56.84)	2/56 (3.57)	7/54 (12.96)	45/95 (47.37)
	T/T	39.75 ± 5.19	2.17 ± 0.45	58/141 (41.13)	41/65 (63.08)	1/43 (2.33)	8/41 (19.51)	31/65 (47.69)
		0.171	0.658	0.126	0.687	0.379[a]	0.010[a.b]	0.320
FOLR1 (rs2071010)	G/G	40.49 ± 4.24	2.23 ± 0.47	170/444 (38.29)	121/199 (60.80)	5/126 (3.97)	13/121 (10.74)	103/199 (51.76)
	A/G	41.50 ± 3.93	2.20 ± 0.41	23/44 (52.27)	16/20 (80.00)	0/16 (0.00)	4/16 (25.00)	11/20 (55.00)
	A/A	43.00 ± 4.00	1.67 ± 0.58	2/5 (40.00)	2/3 (66.67)	1/3 (33.33)	0/2 (0.00)	2/3 (66.67)
		0.362	0.112	0.195	0.188	0.137[a]	0.186[a]	0.929[a]

Note. IVF outcomes of recipients grouped by genotype. Age (in years) and number of embryo transferred are represented by mean and standard deviation (SD) per group. NºET=Number of embryos transferred; P value were calculated by Chi-squared or Fisher's exact tests. [a] Fisher's exact test. [b] *P* value < 0.05. [c] Analysis of the variance (ANOVA)
